# Elevated sweat chloride test: is it always cystic fibrosis?

**DOI:** 10.1186/s13052-021-01060-1

**Published:** 2021-05-14

**Authors:** C. Cimbalo, A. Tosco, V. Terlizzi, A. Sepe, A. Castaldo, L. Salvadori, V. Raia

**Affiliations:** 1 Department of Translational Medical Sciences, Cystic Fibrosis Center, University Federico II, Naples, Italy; 2 Department of Paediatric Medicine, Cystic Fibrosis Centre, Anna Meyer Children’s University Hospital, Florence, Italy

**Keywords:** Sweat chloride test, Cystic fibrosis, False positive, Celiac disease, Klinefelter syndrome

## Abstract

**Background:**

The sweat chloride test (ST) is the gold standard for cystic fibrosis (CF) diagnosis in symptomatic patients, within the newborn screening and in the follow-up of CF patients during molecular therapies. However, false positives have been reported in patients with different diseases. We describe and discuss 4 cases due to different clinical conditions in which we recorded false positive ST, and the test remained altered for a period of varying length.

**Cases presentation:**

Case 1: Eight months old female child suffering from constipation, recurrent vomiting and failure to thrive, family history of recurrent pancreatitis without mutations in the *PRSS1* and *SPINK1* genes. Both ST and fecal elastase were altered although no *CFTR* gene mutations were found. Due to rapid clinical deterioration, celiac disease was suspected and diagnosed by laboratory tests and intestinal biopsy. After 2 weeks of gluten-free diet ST and fecal elastase normalized.

Case 2: 14 months old male suffering from bilateral renal dysplasia, episodes of metabolic alkalosis, recurrent respiratory infections and recurrent vomiting. The child had more ST positives, but no *CFTR* mutations were found. During follow-up, he developed sensorineural hearing loss and an atrial septic defect was found. Finally, a diagnosis of Klinefelter was made, but the ST normalized several years later.

Case 3 and 4: Two boys with stubborn constipation and fecal occlusion treated with Poly Ethylene Glycol (PEG) with salts showed pathological ST. The test returned normal a few days after stopping treatment.

**Conclusions:**

We hypotesized the possible causes of ST alteration in these conditions: in celiac disease it could be due to a transient dysregulation of the aquaporins, rapidly reversed by the diet; in Klinefelter, it may be due to stable pubertal hypoandrogenism; while, the PEG formulation itself contains salts that can temporarily alter ST.

## Background

Cystic fibrosis (CF) is the most common life-threatening inherited disease among Caucasians. More than 2000 variants in the *cystic fibrosis transmembrane conductance regulator* (*CFTR*) gene have been described to date, although the functional consequences have been defined only for several hundred variants [1]. However, despite the increase of sensitivity of molecular analysis [2], the sweat test (ST) remains the gold standard for diagnosis of CF [3]. Furthermore, it is one of the diagnostic tools used in newborn screening (NBS) programs [4] in case of elevated immunoreactive-trypsinogen (IRT) and one of the main biomarkers of efficacy, in terms of improvement of CFTR protein expression/function, in patients treated with the new modulators.

Typically, sweat chloride levels decrease during the first year of life and then increase [5]. Furthermore, a greater intraindividual variability of ST has been reported in patients carrying mutations with varying clinical consequences [6]. Finally, the sweat chloride may be normal in CF patients carrying some CFTR causing mutations with residual function, such as 3849 + 10 kbC > T [7].

False negative or false positive results may be due to inadequate sweat stimulation, incorrect collection and sample handling or inaccurate specimen analysis [8]. For this reasons ST should be performed only by experienced technicians using standardized protocols and quality control programs [9] and should be delayed if the patient is acutely dehydrated, oedematous or receiving corticosteroids or mineralcorticoids, to avoid false positive results [10]. Furthermore, a variety of diseases other than CF can be associated with elevated sweat chloride) even if for most conditions only few cases have been described [10–12], and few data are available to define if this alteration is transient or persistent. Although many of these disorders have clinical manifestations different from CF, the risk of misdiagnosis is still present [13]. Thus, in all patients with altered ST it is mandatory to know and exclude the main causes of a false positive result, particularly in patients that have symptoms that overlap between CF and other diseases. The aim of this paper is to describe four cases of false positive ST affected by other than CF clinical conditions, underlining the importance of a correct interpretation of ST results according to patients’ clinical features and other laboratory parameters (including molecular testing), to allow the correct diagnosis. We also describe the persistence of positive ST, and discuss the causes of altered test in the different conditions.

All the four patients described had not undergone NBS for CF. Sweat chloride testing was performed twice according to approved guidelines [8, 14]. Sweat stimulation and collection was performed using the Gibson-Cooke method [15]. The health care professional involved in ST performing had specific and adequate expertise. The laboratory participates to the Italian proficiency testing since 2014 [9]. Sweat testing was performed only if the following conditions were satisfied: no dehydration, no eczema on the site of stimulation, no edema, no systemic steroid therapy from at least 4 days, no oxygen therapy, according to standard guidelines [8, 14]. Molecular analysis for CF was performed testing the most frequent *CFTR* mutations followed by the whole *CFTR* gene sequencing [16].

## Cases presentation

### Case 1

Eight months old female referred for constipation, recurrent vomiting since the age of 2 months, lack of appetite and failure to thrive since weaning in February 2014. Family history was positive for chronic pancreatitis and negative for celiac disease (CD) and CF. At admission she was malnourished, with anthropometric parameters below normal values. Blood analysis showed Hemoglobin (Hb) 8.6 g/dL, MCV 58.4 fL, AST 457 U/L, with all other routine biochemical blood parameters in the normal range. On the basis of family history, molecular analysis for *PRSS1* and *SPINK1* genes were performed and resulted negative. To rule out CF, we requested ST (Cl 61 mmol/L), fecal elastase (129 μg/gr feces; normal value 200–500 μg/g feces) and an extensive *CFTR* gene sequencing (detection rate 96%) that detected no mutations. When ST was performed the state of hydration of the patient was normal. Two days later her clinical conditions and laboratory parameters got worse, i.e., Hb 7.7 g/dL, AST 521 U/L. Thus, on the basis of vomiting, anorexia, failure to thrive, increased level of liver enzymes and unspecified anemia, CD was suspected and confirmed by laboratory tests (Anti-Transglutaminase IgA > 200 U/mL, anti-endomysium antibodies positive, anti-deaminated gliadin IgA and IgG > 240 U/mL) and esophagogastroduodenoscopy that documented total villous atrophy. Gluten-free diet was started and a prompt improvement of both clinical conditions and blood values (Hb 7,9 g/dL, AST 190 U/L) was observed, as well as the anthropometric parameters. At discharge, fecal elastase was normal (387 μg/gr feces) and ST resulted in the normal range (Cl: 22 mmol/L).

### Case 2

Male patient referred at the age of 14 months for failure to thrive, metabolic alkalosis and renal insufficiency due to bilateral renal dysplasia in 2003. The child suffered from recurrent respiratory infections (chronic airway colonization by *H. influentae* and intermittent colonization by *S. maltophilia* and *P. aeruginosa*) and frequent vomiting episodes. Sweat test was pathological (i.e., Cl: 122 mmol/L). While, *CFTR* gene analysis (detection rate 96%) detected no mutations. Pancreatic status was normal (fecal elastase 413 μg/gr feces). During the follow up lasting 10 years, the patient developed bilateral sensorineural hearing loss and a small atrial septal defect was found in absence of signs and symptoms suggestive of CF. Nevertheless, ST persistently resulted > 60 mEq/L (ranging 92 to 110 mmol/L). All the ST tests were performed in a normal state of hydration. The main causes of false positive ST were ruled out. Based on clinical features, further genetical investigation detected a karyotype suggestive of Klinefelter Syndrome (KS) (47, XXY) when he was 8 years old. The sweat test normalized only several years later.

### Cases 3 and 4

The patient #3 was referred at the age of 11 years old for recurrent abdominal pain and constipation in presence of a good nutritional status. Family history was positive for colorectal polyposis, colorectal adenocarcinoma and type 2 diabetes mellitus, while it was negative for CF. At the age of 10 years old he complained of recurrent abdominal pain under umbilicus. For this reason, the following analysis were performed: H2 breath test, parasitological stool examination, laboratory tests for celiac disease, antinuclear antibodies, faecal calprotectin, urinalysis and abdominal ultrasound examination; all with normal results. After few months he was admitted twice at the emergency room for worsening of symptoms; in those occasions the ultrasound abdominal study showed coprostasis and was started a treatment with a PEG (polyethylene glycol) formulation with salt (sodium bicarbonate, sodium chloride and potassium chloride). The ST was performed and showed pathological values (i.e., Cl: 65 mmol/L). No mutations were found at the molecular analysis of *CFTR* gene. Therefore, the therapy with PEG was suspended and after a week the ST was normal (Cl: 12 mmol/L).

The patient #4 was referred at the age of 17 years old for history of constipation from the first month of life, an episode of sub-ileus at 5 years and intermittent therapy with faecal softening and enema that reduced the symptoms. At the age of 16 years, he was hospitalized for intestinal occlusion and CT showed a rectal fecaloma and colic overextension, so he immediately started the evacuating enemas; at the end of the hospitalization he was discharged with the indication to a colonoscopy, therapy with PEG and the evaluation in a specialistic center. He came to our attention and we performed analysis for CD, fecal calprotectin, total antibodies and routines biochemistry that resulted normal; ST was pathological (Cl: 60 mmol/L). No mutations were found at the molecular analysis of *CFTR* gene. Therefore, the therapy with PEG was suspended and after a week the ST was normal (Cl: 12 mEq/L).

## Discussion and conclusions

We describe four cases of false positive ST in children suffering from CD, KS or who were taking PEG with salt for chronic constipation. We performed the ST in the same laboratory, thus excluding the lack of standardization between different labs [10] and tested sweat twice in all cases to rule out the possibility of sample collection errors [14].

It is known that ST may give false negative or positive results with a rate ranging 10 to 15% [12]. We retrospectively analysed data about the ST collected at our CF centre (that acts as regional reference centre for Campania Region, about 6 million of inhabitants) between 2000 and 2019: during this period about 10,000 tests were made and we identified 7% false positives (data not shown). A complete knowledge of the factors causing false positive results is crucial in order to interpret data in a clinically appropriate manner allowing a correct diagnosis, but recent studies on large number of patients with other disorders lack. Furthermore, no studies have been performed so far to establish if the false positive results in other than CF diseases are stable or transient. For some diseases, mechanisms are well known that may cause the false positive ST, thus causing a false positive result, e.g., patients affected by endocrine diseases or during therapies [12, 17]. In other cases, alterations that may cause the increase of sweat chloride are still poorly known. Celiac disease and CF share several clinical manifestations and a higher prevalence of CD among CF patients is known [18]. Celiac disease may cause a false positive ST [8, 12], but the reason is still unknown. Previous studies reported that ST is elevated in patients with malnutrition [19]. Both CFTR and the basolateral sodium potassium ATPase (Na/K ATPase) require ATP for their activity and ATP levels are reduced in tissues from malnourished rats [20]. Furthermore, it was hypothesized that malnutrition causes a reduction in alveolar epithelial sodium and chloride transport implying a reduction in the function of alveolar epithelial CFTR and ENaC channels. Nevertheless, no changes in CFTR expression were observed in CD, even if overexpression of CFTR could be due to the hypertrophy and hyperplasia of crypt of small intestinal mucosa typical of CD [21]. Likewise, recently the effects of CD on aquaporins and on the expression of solute carriers has been described [21], thus, such effect may also explain the frequent occurrence of false positive ST in CD. Moreover, recent literature demonstrated a complex role of CFTR dysfunction in the immunopathology of CD [22]. This evidence also supported the close link between CF and CD and agrees with the normalization of ST in CD after diet restriction therapy.

Klinefelter syndrome was regarded as one of the possible causes of transient chloride elevation in sweat in few cases [23]. Some cases of Klinefelter syndrome associated to CF have been described in adult patients [24, 25], but they had been described before the discovery of *CFTR* gene, thus the diagnosis of CF may be questioned. The most recent American guidelines excluded KS among the possible causes of chloride temporary elevation [8]. In male patients with persistent or transient elevated ST with negative *CFTR* gene analysis and in absence of CF typical symptoms, KS diagnosis has to be considered, performing karyotype analysis. The causes that may explain the increase of ST in KS are still unknown: we may only speculate, according to literature evidences, that the higher body size of these subjects and the severe deficiency of androgens [26, 27] during puberty may influence the activity of sweat glands causing a persistent SC alteration. Finally, for the two cases treated with PEG, it is well known that some formulations of the drug contain salts and thus they may alter the ST. The rapid normalization of the ST after the drug suspension indicate that in the routine setting all patients treated with such drugs should suspend it before testing. In our experience the ST normalized 1 week after suspension, but more experience is needed regarding the timing to normalize sweat chloride after PEG treatment. However, it is possible also to use PEG without electrolytes. Thus, we suggest adding such indication in the future guidelines for ST.

To conclude: in all patients with altered ST it is mandatory to know and exclude the main causes of a false positive result, particularly in patients that have symptoms that overlap between CF and other diseases. Furthermore, the results of molecular testing and clinical alterations must be considered in each patient in addition to SC. Finally, a systematic study must be performed to better define all the diseases that may cause false positive ST, also clarifying the duration of ST alteration.

## Data Availability

All clinical data and material are available in our Unit.

## Abbreviations

CF: Cystic fibrosis
CFTR: CF transmembrane regulator
ST: Sweat test
NBS: Newborn screening
IRT: Immunoreactive trypsinogen
CD: Celiac disease
KS: Klinefelter syndrome
